# The association between metabolic syndrome and vascular endothelial dysfunction in adolescents

**DOI:** 10.3892/etm.2013.1055

**Published:** 2013-04-09

**Authors:** YING WEI, GELI LIU, JINGYAN YANG, RONGXIU ZHENG, LIHONG JIANG, PENGLI BAO

**Affiliations:** Department of Pediatrics, General Hospital of Tianjin Medical University, Tianjin 300052, P.R. China

**Keywords:** metabolic syndrome, vascular endothelium functional lesion, von Willebrand factor, plasminogen activator inhibitor-1

## Abstract

The aim of this study was to investigate the association between metabolic syndrome (MS) and vascular endothelial cell dysfunction (ECD) in adolescents. Sixty obese pediatric inpatients at the General Hospital of Tianjin Medical University from February 2011 to February 2012 were included. Among these, 30 patients were obese and 30 patients were diagnosed with MS. Thirty healthy subjects were randomly selected as the control group. A series of indices, including height, body weight, waist circumference, hip circumference, waist/hip circumference and body mass index (BMI), as well as total cholesterol (TC), triglyceride (TG), low-density lipoprotein (LDL) and high-density lipoprotein (HDL) levels were evaluated. von Willebrand factor (vWF) and plasminogen activator inhibitor-1 (PAI-1) levels were determined using an enzyme linked immunosorbent assay (ELISA). Correlation analysis between height, body weight, waist circumference, hip circumference, waist/hip circumference, BMI, TC, TG, LDL, HDL and PAI-1, as well as vWF was performed. Significant increases of vWF and PAI-1 levels were observed in the MS group compared with the control group (P<0.05). For the adolescents in the obese group, a significant increase of PAI-1 level was observed compared with the control group (P<0.05). No significant difference was observed between the vWF levels in the obese and control groups. PAI-1 was positively associated with BMI, waist circumference, waist/hip circumference, TC, TG, LDL, fasting plasma glucose (FPG), fasting insulin (FINS), systolic blood pressure (SBP) and diastolic blood pressure (DBP), respectively (P<0.05). In addition, PAI-1 was negatively associated with HDL levels (P<0.05). PAI-1 and vWF may be used as biomarkers for the diagnosis of ECD. ECD in individuals with MS may be associated with obesity, blood fat, blood sugar and blood pressure. FPG, TC and TG may be risk factors for ECD.

## Introduction

Metabolic syndrome (MS), also known as syndrome X, refers to an insulin resistance syndrome characterized by glucose intolerance, insulin resistance, central obesity, dyslipidemia and hypertension, which are all documented risk factors for cardiovascular disease (CVD) (1). There has been a significant increase in the number of patients with MS worldwide, which is associated with the global epidemic of obesity and diabetes. Therefore, there is an urgent need for strategies to prevent the emerging global epidemic.

Vascular endothelial function is considered to be associated with MS in children (2). However, the mechanism of the association remains unclear. von Willebrand factor (vWF) and plasminogen activator inhibitor-1 (PAI-1) are considered two of the major biomarkers for vascular endothelium function. Previous studies have indicated that vWF levels increase in patients with hypertension (3) and that plasma vWf is correlated with coronary risk (4) and an adverse prognosis (5). In addition, vWF levels have been reported to increase with increasing insulin levels (6), which suggests a correlation between insulin resistance and MS. Nevertheless, few data on vWf in relation to the cumulative number of components of MS are available. The links between PAI-1, MS and obesity were established a number of years ago (7). However, few studies have been performed to investigate the potential role of PAI-1 in adolescents with MS. The aim of the current study was to investigate the levels of vWF and PAI-1 in children with MS.

## Patients and methods

### Patients

Sixty obese pediatric inpatients at the General Hospital of Tianjin Medical University from February 2011 to February 2012 were included in this study. Of these, there were 30 patients with obesity (15 females and 15 males) with an average age of 11.40±1.43 years and 30 patients with diagnosed MS (13 females and 17 males) with an average age of 11.62±1.32 years. Thirty healthy subjects were randomly selected as the control group (15 females and 15 males) with an average age of 11.23±1.04 years.

The diagnostic criteria for obesity were set according to those previously described (8). The diagnosis of MS in children was performed according to the criteria set by International Diabetes Federation (IDF) (9).

For the physical examination, body weight and height were determined in the morning in a fasting state. Waist circumference, hip circumference and abdominal circumference were determined according to the previous descriptions.

### Glycolipid metabolism index

Venous blood was collected from the patients in a state of fasting for 8 h. The blood glucose levels were determined using the glucose oxidase method with kits purchased from Biosino Bio-Technology & Science Inc. (Beijing, China). The level of insulin was evaluated using a kit purchased from the China Institute of Atomic Energy (Beijing, China). Glycolipid analysis was performed using a Hitachi 7170 automatic biochemistry analyzer (Tokyo, Japan).

### Determination of vWF and PAI-1

The levels of vWF and PAI-1 were analyzed using the standard kits purchased from Beijing Gersion Bio-Technology Co., Ltd. (Beijing, China). All procedures were performed strictly according to the manufacturer’s instructions.

### Statistical analysis

All data are presented as mean ± standard deviation. One-factor analysis of variance was conducted for intergroup comparisons. Correlation analysis was performed using Pearson correlation analysis. Multiple step-wise regression analysis was performed for the evaluation of PAI-1 as a risk factor. P<0.05 was considered to indicate a statistically significant difference.

## Results

### Expression of PAI-1 and vWF

The level of PAI-1 in the MS group demonstrated a marked increase compared with that of the control group (P<0.01). In addition, the level of PAI-1 in the subjects in the obese group demonstrated a significant increase compared with that of the control group (P<0.01). No statistical difference was noted in the level of PAI-1 between the obese and MS groups (P>0.05; Table I). The level of vWF in the MS group was observed to be markedly higher than that of the control group (P<0.01). Additionally, no significant difference was noted in the level of vWF in the obese group compared with the control group (P>0.05). A significant difference was noted in the level of vWF between the MS and obese groups (P<0.05).

### Correlation analysis of PAI-1 with various parameters

In this study, the correlations between PAI-1 and body weight, body mass index (BMI), waist circumference, waist/hip circumference and hip circumference, as well as total cholesterol (TC), triglycerides (TG), low-density lipoprotein (LDL), fasting plasma glucose (FPG), fasting insulin (FINS), systolic blood pressure (SBP), diastolic blood pressue (DBP) and high-density lipoprotein (HDL) levels were analyzed respectively. The results indicated that PAI-1 was positively associated with BMI, waist circumference, waist/hip circumference, TC, TG, LDL, FPG, FINS, SBP and DBP, respectively (P<0.05). In addition, PAI-1 was identified to be negatively associated with HDL (P<0.05, Table II).

### Multiple step-wise regression analysis

Table III shows the step-wise regression analysis of PAI-1 with various parameters. The results indicated that FPG, TC and TG affect the level of PAI-1 independently.

## Discussion

Cases of obesity and overweight have been frequently reported in children and adolescents in China and the incidence has increased to a new high. The first diagnostic criteria for MS in children and adolescents were set in 2007 by the IDF, and were created based on the diagnostic criteria for adults (9). A consultation group for the World Health Organization (WHO) consultation proposed a set of criteria for MS (10). Then, the National Cholesterol Education Program’s Adult Treatment Plan III and the European Group for the Study of Insulin Resistance formulated definitions (1). These definitions stated that glucose intolerance, obesity, hypertension and dyslipidemia should be applied for the diagnosis of MS. These definitions laid the foundation for the clinical diagnosis of MS in children and adolescents. Additionally, they are of prime importance for the prevention and treatment of CVD and type II diabetes mellitus.

MS, a major cause of multiple cardiovascular risks, has been reported to be associated with CVD. Previous studies indicated that the risk factors of atherosclerotic heart disease in adults may be related to the prevalence of an MS phenotype (11,12). Vascular endothelial cells, which form the inner lining of major blood vessels, play an important role in the modulation of vasomotion, the integrity of vessel walls, avoiding the aggregation of platelets and the endocrine function of the organs. Various factors, including risk factors of CVD, may lead to disorders of vascular endothelial cells (VECs), which may cause endothelial cell dysfunction (ECD). A number of studies have demonstrated that ECD is the initial pathological and physiological change of CVD (13–15). The emergence of ECD may be considered to be an indicator of CVD. The identification and treatment of ECD for MS patients is crucial in the prevention, treatment and outcomes for CVD. Thus, preventing ECD has been considered as a method for the treatment of MS. The integrity of the vessel wall is crucial for the normal circulation of blood. When damage to the vascular surface occurs, vWF binds rapidly to exposed subendothelial structures and enables platelet arrest from fast-flowing blood through the interaction of its A1 domain with the platelet glycoprotein Ibα (16). PAI-1, a serine protease inhibitor, functions as the principal inhibitor of tissue plasminogen activator and urokinase (17). PAI-1 inhibits thrombin activity in the presence of vitronectin. In addition, PAI-1 inhibits factor Xa, which may reduce thrombin formation. PAI-1 also effectively inhibits the activity of tissue-type plasminogen activator (tPA).

The association of MS and ECD in adults has been well-defined. However, few studies have been performed to investigate their correlation in children and adolescents. At present, PAI-1 and vWF are considered the major biomarkers for the function of endothelial cells. In our study, the expression levels of PAI-1 and vWF in serum were determined to investigate the occurrence of ECD. The results revealed significant increases in the levels of PAI-1 and vWF in the MS group compared with the control group (P<0.01). We considered that PAI-1 and vWF may be used for the early identification of MS and ECD. In the obese group, the expression levels of vWF were not observed to be significantly different from those of the control group (P>0.05). However, the expression levels of PAI-1 demonstrated a marked increase in these patients compared with the control group (P<0.05). Huang *et al* (18) reported that children with obesity presented symptoms of ECD. Thus, the prevention of ECD should not be limited to children with MS. Instead, those with obesity should receive more attention to prevent the occurrence of MS and ECD.

Previous studies have indicated that the incidence of MS in children is increasing, particularly in those with obesity (19). The clinical symptoms of MS in children include obesity (particularly abdominal obesity), dyslipidemia (increases in TG, TC and LDL levels, and reductions in HDL levels), hypertension, hyperinsulinism and type II diabetes (20). Currently, obesity and IR are considered the major causes of MS (21). Additionally, these factors are directly associated with MS and CVD. MS is associated with multiple cardiovascular risk factors; however, the precise pathophysiology of this clinical syndrome is not clear. Previous studies proposed that endothelial damage and/or dysfunction may be a crucial component of MS (22,23). Another study demonstrated the association between plasma indices of endothelial damage and/or dysfunction, including vWF, with features of MS (24). A temporal correlation (with cause preceding effect) has not been defined.

According to the results of the current study, a positive correlation was identified between PAI-1, body weight, BMI, waist circumference, waist/hip ratio, TC, TG, LDL, FPG, FINS, SBP and DBP, respectively (P<0.05). A negative correlation was identified between PAI-1 and HDL (P<0.05). Based on this, we considered that MS plus ECD in children may be associated with obesity (particularly abdominal obesity), blood fat, blood sugar and blood pressure. Moreover, multiple step-wise regression analysis indicated that FPG, TC and TG were the major causes of MS plus ECD in children. A previous study indicated that increases in blood sugar levels induce the increase of interleukin (IL)-6, IL-12, IL-1 and tumor necrosis factor (TNF)-α, which may induce CVD in an early stage (25). In addition, Myers *et al* (26) reported that a high blood sugar-induced vascular endothelial lesion synergizes the activation of lipid, while the increase of blood fat exacerbates the vascular endothelial cell damage caused by elevated blood sugar. In conclusion, our results indicated that PAI-1 and vWF may be used as biomarkers for the diagnosis of ECD. Furthermore, MS patients presented symptoms of ECD may be associated with obesity, blood fat, blood sugar and blood pressure.

## Figures and Tables

**Table I:** Levels of PAI-1 and vWF in serum.

Group	N	PAI-1 (ng/ml)	vWF (U/l)
MS group	30	106.05±5.11	106.82±21.67
Obese group	30	105.53±2.02	94.37±20.79
Control group	30	102.35±4.43	91.32±17.40
F-value		7.246	5.044
P-value (1:2)		0.623	0.018
P-value (1:3)		0.001	0.004
P-value (2:3)		0.003	0.556

Data are presented as mean ± standard deviation. PAI-1, plasminogen activator inhibitor 1; vWF, von Willebrand factor; MS, metabolic syndrome. 1, MS group; 2, obese group; 3, control group.

**Table II:** Correlation analysis between PAI-1 and various parameters.

Item	RS	P-value
Body weight	0.489	0.006
BMI	0.534	0.002
Waist circumference	0.423	0.02
Hip circumference	0.216	0.251
Waist/hip circumference	0.586	0.001
TC	0.663	<0.01
TG	0.645	<0.01
HDL	−0.533	0.002
LDL	0.419	0.021
FPG	0.419	0.021
FINS	0.405	0.026
SBP	0.492	0.006
DBP	0.409	0.025

PAI-1, plasminogen activator inhibitor 1; BMI, body mass index; TC, total cholesterol; TG, triglycerides; HDL, high-density lipoprotein; LDL, low-density lipoprotein; FPG, fasting plasma glucose; FINS, fasting insulin; SBP, systolic blood pressure; DBP, diastolic blood pressure.

**Table III:** Multiple step-wise regression analysis.

Variable	Regression of coefficient	Standard deviation	P-value
FPG	2.223	0.927	0.029
TC	2.298	0.891	0.020
TG	2.152	0.740	0.010

FPG, fasting plasma glucose; TC, total cholesterol; TG, triglycerides.
